# Treatment With Cladribine Selects IFNγ+IL17+ T Cells in RRMS Patients – An *In Vitro* Study

**DOI:** 10.3389/fimmu.2021.743010

**Published:** 2021-12-14

**Authors:** Minodora Dobreanu, Doina Ramona Manu, Ion Bogdan Mănescu, Manuela Rozalia Gabor, Adina Huţanu, Laura Bărcuţean, Rodica Bălaşa

**Affiliations:** ^1^ Department of Immunology, Centre for Advanced Medical and Pharmaceutical Research, “George Emil Palade” University of Medicine, Pharmacy, Science and Technology, Târgu Mureș, Romania; ^2^ Clinical Laboratory, County Emergency Clinical Hospital, Târgu Mureș, Romania; ^3^ Department of Laboratory Medicine, “George Emil Palade” University of Medicine, Pharmacy, Science and Technology, Târgu Mureș, Romania; ^4^ Department of Management and Economy, “George Emil Palade” University of Medicine, Pharmacy, Science and Technology, Târgu Mureș, Romania; ^5^ Neurology 1 Clinic, County Emergency Clinical Hospital, Târgu Mureș, Romania; ^6^ Department of Neurology, “George Emil Palade” University of Medicine, Pharmacy, Science and Technology, Târgu Mureș, Romania

**Keywords:** relapsing-remitting multiple sclerosis (RRMS), cladribine, T cells, IFNγ+IL17+ cells, Th17.1 cells, *in vitro*, disease modifying drug (DMD)

## Abstract

**Background:**

Multiple sclerosis (MS) is an incurable autoimmune disease mediated by a heterogeneous T cell population (CD3+CD161+CXCR3−CCR6+IFNγ−IL17+, CD3+CXCR3+CCR6+IFNγ+IL17+, and CD3+CXCR3+IFNγ+IL17− phenotypes) that infiltrates the central nervous system, eliciting local inflammation, demyelination and neurodegeneration. Cladribine is a lymphocyte-depleting deoxyadenosine analogue recently introduced for MS therapy as a Disease Modifying Drug (DMD). Our aim was to establish a method for the early identification and prediction of cladribine responsiveness among MS patients.

**Methods:**

An experimental model was designed to study the cytotoxic and immunomodulatory effect of cladribine. T cell subsets of naïve relapsing-remitting MS (RRMS) patients were analyzed *ex vivo* and *in vitro* comparatively to healthy controls (HC). Surviving cells were stimulated with rh-interleukin-2 for up to 14days. Cell proliferation and immunophenotype changes were analyzed after maximal (phorbol myristate acetate/ionomycin/monensin) and physiological T-cell receptor (CD3/CD28) activation, using multiparametric flow cytometry and xMAP technology.

**Results:**

*Ex vivo* CD161+Th17 cells were increased in RRMS patients. *Ex vivo* to *in vitro* phenotype shifts included: decreased CD3+CCR6+ and CD3+CD161+ in all subjects and increased CD3+CXCR3+ in RRMS patients only; Th17.1 showed increased proliferation vs Th17 in all subjects; CD3+IL17+ and CD3+IFNγ+IL17+ continued to proliferate till day 14, CD3+IFNγ+ only till day 7. Regarding cladribine exposure: RRMS CD3+ cells were more resistant compared to HC; treated CD3+ cells proliferated continuously for up to 14 days, while untreated cells only up to 7 days; both HC/RRMS CD3+CXCR3+ populations increased from baseline till day 14; in RRMS patients vs HC, IL17 secretion from cladribine-treated cells increased significantly, in line with the observed proliferation of CD3+IL17+ and CD3+IFNγ+IL17+ cells; in both HC/RRMS, cladribine led to a significant increase in CD3+IFNγ+ cells at day 7 only, having no further effect at day14. IFNγ and IL17 secreted in culture media decreased significantly from *ex vivo* to *in vitro*.

**Conclusions:**

CD3+ subtypes showed different responsiveness due to selectivity of cladribine action, in most patients leading to *in vitro* survival/proliferation of lymphocyte subsets known as pathogenic in MS. This *in vitro* experimental model is a promising tool for the prediction of individual responsiveness of MS patients to cladribine and other DMDs.

## 1 Introduction

Multiple Sclerosis (MS) is an incurable autoimmune disease affecting the central nervous system (CNS) and is associated with T cell-mediated immunopathology during all phases of the disease. Numerous genetic and pathological studies performed in the last decade point towards T and B cells, as essential players in MS pathogenesis. MS affects only the CNS, as an argument that T and B cells are recruited by CNS specific antigens (1). Activated T cells (CD3+) are capable of mounting an autoimmune response against myelin components, penetrating the blood-brain barrier, proliferating and subsequently secreting pro-inflammatory cytokines. These cytokines stimulate microglia, macrophages, astrocytes and B cells, resulting in demyelination and neurodegeneration (2, 3).

In the peripheral blood of MS patients, a large number of heterogeneous, IL17-producing CD3+ cells have been identified, especially during disease exacerbation (4, 5). In addition to a Th17 phenotype (CD3+CD4+CD161+CCR6+) characterized by IL17 production, and a classical Th1 phenotype (CD3+CD4+CXCR3+) which is associated with production of IFNγ (6, 7), a heterogeneous CD3+ cell population has been shown to be implicated in MS pathogenesis: these cells express phenotypic markers of both Th1 and Th17 cells, but in varying levels and combinations, and also exhibit a mixed profile, characterized by a CCR6+CXCR3+ immunophenotype and concomitant secretion of IL17A and IFNγ (8–11). Furthermore, both IL17-producing CD3+CD4+ and CD3+CD8+ cells were highlighted in the peripheral blood and cerebrospinal fluid of MS patients in early stages of the disease (12–14). A clonal expansion of CD3+CD8+ cells takes place within the brain inflammatory microenvironment at the onset of disease, beginning with IL17 production (15–18). The number of CD3+CD8+ cells surpasses the number of CD3+CD4+ cells in the inflammatory infiltrate of active demyelinating plaques regardless of disease stage, duration or progression (19, 20).

Relapsing-remitting MS (RRMS) is the most common clinical form of MS, accounting for about 85% of all MS cases (21). RRMS patients may benefit from treatment, but individual response to a given therapy and the occurrence of adverse events are largely unpredictable. Thus, many patients need to change several drugs in order to stabilize their disease. Current therapy consists mainly of disease-modifying drugs (DMDs) with immunomodulatory or immunosuppressive action, targeting inflammation in patients in order to reduce the relapse rate and to delay progression (22, 23).

Cladribine was first synthesized in 1972, but only approved by the FDA in the early ‘90s for the treatment of certain leukaemias (24). In 2010, cladribine was approved for RRMS in Russia and Australia, but was later removed (24). Based on clinical data from the CLARITY, CLARITY-EXT, ORACLE-MS, and PREMIERE studies, in 2017 cladribine was approved in the EU for the treatment of active RRMS (24). In 2019, cladribine was also approved by the FDA for active RRMS and, as of July 2020, it is approved in more than 75 countries (24). This synthetic deoxyadenosine analog is a prodrug, which is incorporated into the DNA of proliferating cells (like activated lymphocytes), causing strand breakage, followed by p53 activation and programmed cell death (apoptosis) through the caspase system (25, 26).

Cladribine acts as a lymphocyte-depleting drug, but preferentially depletes memory B cells: in MS patients, Cladribine can deplete 40-50% of total T cells, and approximately 80% of total B cells (27, 28), but informations on the efficacy of this drug in RRMS are scarce (29). Moreover, in some patients cladribine is not able to stop the progression of disease and induce a long-lasting remission. Hence, methods able to predict responsiveness to cladribine would be very useful in the clinical setting, allowing for the early identification of cladribine (non-)responders and assisting clinicians in proper treatment selection. however, to date, little progress has been made in personalized therapy and response prediction in MS. It is essential to identify non-responders to a therapeutic molecule before the appearance of advanced neurological lesions. Additionally, side effects of immunosuppressants or monoclonal antibodies in MS patients, as well as specific risk-benefit ratios, should be established from the earliest stages of patient management. The aim of our study was to assess an experimental model that can characterize the changes in surviving T-cell subpopulations following cladribine exposure in order to create an algorithm of responsiveness to DMDs that could assist clinicians in choosing the best therapy for each patient, avoiding additional CNS lesions, disability progression, and unnecessary costs. Cytotoxic and immunomodulatory effects of cladribine on T cells from naïve RRMS patients were studied by tracking changes in phenotypic markers of aggressivity, such as the T cell surface markers CXCR3, CCR6 and CD161, as well as the secretion of IFNγ and IL17. Such experimental models may prove useful for establishing a personalized approach to therapy in MS patients.

## 2 Materials and Methods

### 2.1 T Cell Isolation and Activation

Our study was approved by the Committee for Ethical Research of the Emergency Clinical County Hospital of Târgu Mureş (decision no. 7,100/2018) and the experiments were performed according to the Declaration of Helsinki principles for experiments involving humans. For T cell isolation, peripheral blood from RRMS naïve patients and healthy donors (healthy controls, HC) was collected in heparinized tubes (Greiner Bio-One, cat. no. 455051). Automated complete blood counts (CBC) were performed using a Sysmex XS-800i hematology analyzer and hsCRP plasma levels were analyzed with a BN ProSpec System using N Latex CardioPhase hsCRP Reagent (Siemens, cat. no. OQIY 21). Samples with more than 10×10^9^ white blood cells/L and/or hsCRP above 3 mg/L were excluded. Peripheral blood mononuclear cells (PBMCs) were isolated by Ficoll-Paque (Sigma, cat. no. H8889) density gradient centrifugation, using a standard operating procedure previously implemented in our laboratory (30), and cryopreserved with 10% DMSO at -140°C, until analysis. PBMCs were then cultured in RPMI-1640 medium containing L-glutamine (Sigma, cat. no. R8758) supplemented with 1% penicillin/1% streptomycin (Sigma A, cat. no. P4333) and 10% fetal bovine serum (FBS; Sigma, cat. no. F7524) at a density of 1-2×10^6^ cells/mL.

*Ex vivo* analysis was performed after short-term maximal stimulation for 4-5 hours with 50 ng/mL phorbol 12-myristate 13-acetate (PMA; Sigma, cat. no. P-8139) and 1 μg/mL ionomycin (Sigma, cat. no. I-0634), in the presence of 0.1 μg/mL monensin (GolgiStop, BD Pharmingen, cat. no. 554724). Surface phenotype and intracellular cytokines were evaluated by multi-parametric flow cytometry analysis. An additional fraction of cells was subjected to physiological TCR activation with soluble NA/LE™ CD3 monoclonal (mAb) antibody (BD, clone HIT3a, cat. no. 555336) at a final concentration of 1 µg/mL, and CD28 mAb (BD, clone 28.2, cat. no. 555725) at a final concentration of 5 µg/mL, for 72 hours. Culture media were harvested for cytokine secretion analysis.

For *in vitro* medium-term analysis, thawed PBMCs were treated with CD3/CD28 mAbs for 72 hours, then transferred into media supplemented with 10 ng/mL recombinant human IL2 (rh-IL2; BD Pharmingen, cat. no. 554603) for a period of either 5 or 10 days. rh-IL2 was removed 20 hours prior to cell re-stimulation on day 7 and 14. The re-stimulations were performed with PMA/ionomycin/monensin for multi-parametric flow cytometric assessment of the phenotypic profile, and with CD3/CD28 mAbs for analysis of cytokine secretion in culture media. Cell culture and activation protocols were previously described by Korsen et al. (31).

**Figure 1:**
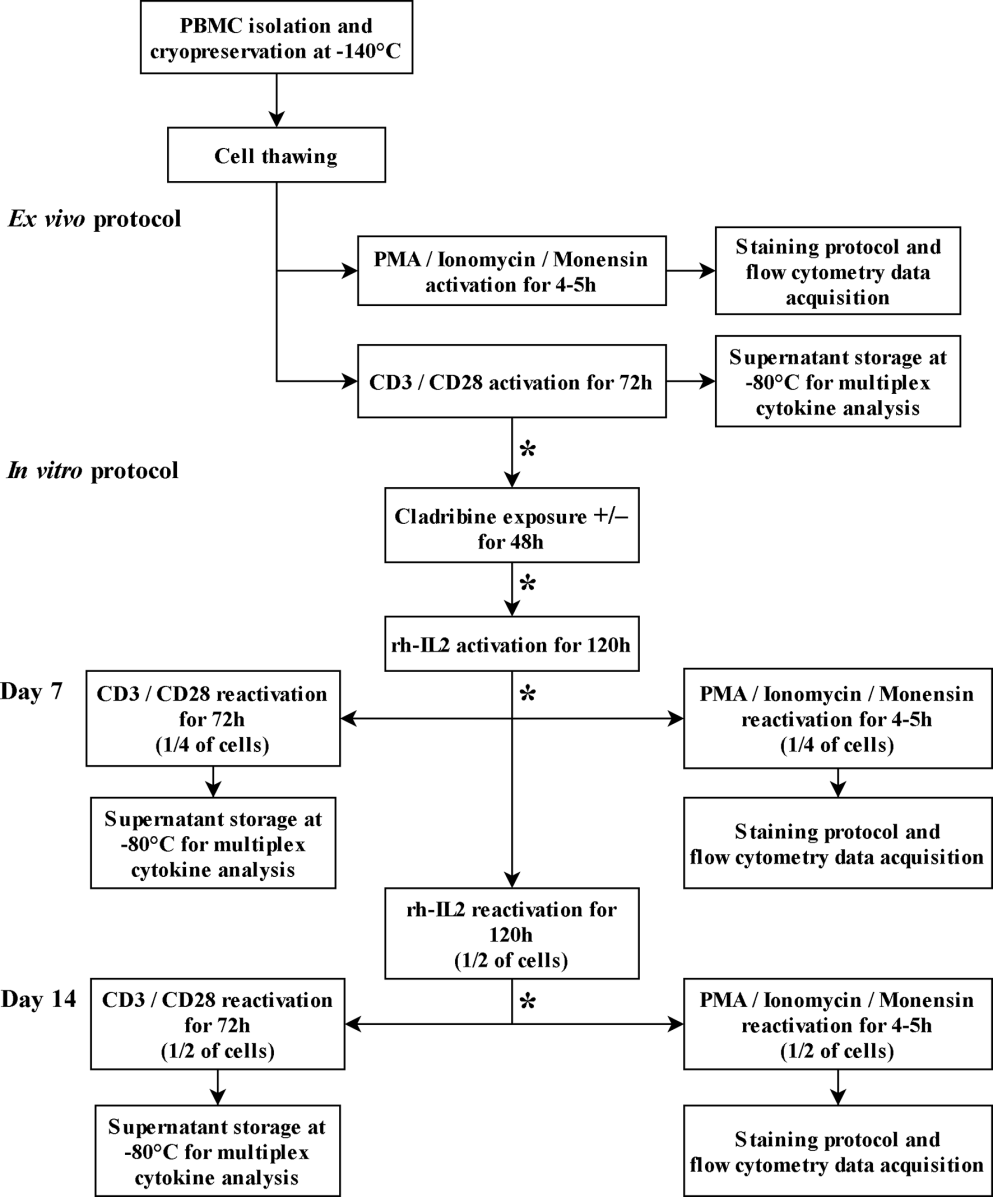
T cell culture and activation protocols. The asterisk (*) between steps indicates cell wash, cell count, and viability assessments.

Details of protocols are described in **Figure 1**.

### 2.2 *Ex Vivo* Versus *In Vitro* Maximally Activated T Cell Phenotypic Profile

**Figure 2:**
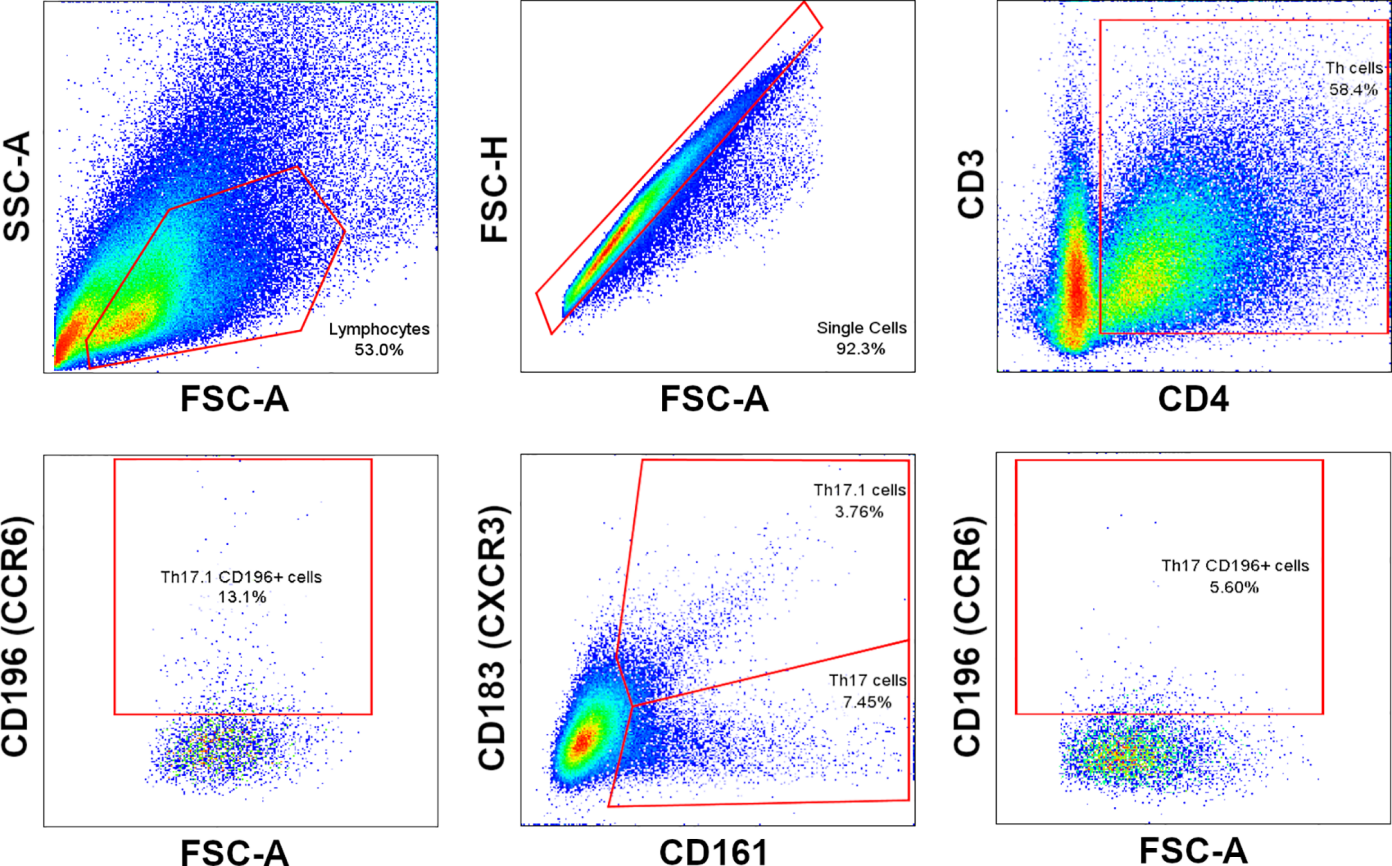
Cell gating strategy to identify Th17 and Th17.1 cell subpopulations.

Phenotypic profile analysis of T cell subsets in *ex vivo* (immediately after thawing cryopreserved cells) and *in vitro* (after maintaining thawed cells in culture for 7 and 14 days) samples consisted of immunostaining for cell surface markers, lineage-specific transcription factors, and intracellular cytokines. The first panel included specific fluorochrome-conjugated antibodies against cell surface biomarkers CD3, CD4, CD161, CXCR3, CCR6 and intracellular cytokines IFNγ and IL17. The expressions of lineage-specific transcription factors TBet and RORγt were measured with a secondary panel of fluorochrome-conjugated antibodies that tagged the same surface markers. The BD FACSAria III cytometer configuration as well as the antibodies used in the protocol are presented in **Table 1**. Data acquisition and analysis were performed using BD FACSDiva™ digital software. A minimum of 30,000 CD3+ events was acquired per sample. Details of cell gating strategy to identify the CD3+CD4+ cell population of interest by flow cytometry are shown in **Figure 2**.

**Table 1:** Parameter specifications and BD FACSAria III cytometer configuration used for data acquisition.

Excitation LASER lines	Fluorochrome	Maximum emission (nm)	Band-Pass filters (nm)	Relative Brightness	Mouse antibody clone	Specificity
Violet (405 nm)	BD Horizon™ BV421	421	450/40	Brightest	B27	Human IFNγ/TBet
	BD Horizon™ BV510	510	530/30	Moderate	UCHT1	Human CD3
Blue (488 nm)	BD Pharmingen™ Alexa Fluor^®^ 488	519	530/30	Moderate	1C6/CXCR3	Human CD183
						(CXCR3)
	BD Pharmingen™ PE	578	575/26	Bright	DX12	Human CD161
	BD Pharmingen™ PE-Cy7™	785	780/60	Brightest	11A9	Human CD196
						(CCR6)
Red (633 nm)	BD Pharmingen™ Alexa Fluor^®^ 647	668	660/20	Bright	N49-653	Human IL17/RORγt
	BD Pharmingen™ Alexa Fluor^®^ 700	719	730/45	Dim	SK3	Human CD4
	Fixable Viability Stain 780	780	780/60	–	–	Cell-surface and intracellular amines

### 2.3 The Cytotoxic Effect of Cladribine

TCR-activated cells were incubated for 48 hours with 10^-7^ M cladribine (Sigma Aldrich, cat. no. C4438) or in the absence of cladribine, as control samples. After the incubation period, the cytotoxic effect of cladribine was immediately assessed by absolute CBC using Sysmex XS 800i Hematology Analyzer, followed by a viability assay using the FACSAria III flow cytometer and Becton Dickinson Horizon™ Fixable Viability Stain 780 (FVS 780, BD cat. no. 565388). Necrotic cells were determined by higher levels of FVS 780, with a 10-20-fold increase in intensity over viable cells.

TCR-activated lymphocytes cultured for 48h with or without cladribine were transferred to a cladribine-free medium with rh-IL2 for up to 7 and 14 days. Lymphocytes were analyzed for viability at day 0 (before culture initiation) and at day 7 or 14, as described above.

The changes in absolute lymphocyte number reflected cell proliferation or depletion in response to the cytotoxic action of cladribine. Survival indexes were established as a ratio between absolute viable cell number without or after cladribine exposure, and initial absolute viable cell number. Proliferation indexes were calculated as a ratio of absolute viable cell number before and after 7 days and 14 days of culture with rh-IL2.

### 2.4 Evaluation of the Immunomodulatory Effect of Cladribine on the Cytokine Secretory Profile of TCR-Activated Cells

After 7-14 days of culturing cells exposed/unexposed to cladribine, lymphocytes were stimulated with PMA/ionomycin/monensin, and analyzed by flow cytometry for immunophenotypic changes. Immunophenotypic shifts were defined as changes in cell surface receptors (CD161, CXCR3, CCR6) and intracellular cytokines (IFNγ and IL17) expression in T cell subpopulations. The immunomodulatory effect of cladribine was quantified by flow cytometry as changes in the percentages of positive cell subpopulations, labeled with the specific antibodies for antigens of interest, as listed in **Table 1**. The same antibody panels and cytometer configurations were used for the assessment of cladribine effect on e*x vivo* versus *in vitro* T cell phenotype.

The ideal method for *in vitro* activation of T cells should mimic the signaling events that occur during physiological activation, and agonistic antibodies against the TCR/CD3 complex with the co-stimulatory molecule CD28 are largely used for this purpose. In our study, the T cell signature of secreted cytokines was analyzed in *ex vivo* and *in vitro* lymphocytes at day 7 and day 14, after re-stimulation with CD3/CD28 mAbs for 72 hours. The supernatant was collected and stored at -80°C until analysis. The secreted cytokine profile associated with TCR-activated cells was measured using xMAP Technology on FlexMap 3D Luminex analyzer and a cytokine panel, including IFNγ and IL17A built with ProcartaPlex™ Multiplex Kits from Invitrogen [Human High-Sensitivity Panel 9-plex, cat. no. EPXS090-12199-901]. Data were acquired and analyzed with xPONENT software for Luminex instruments.

### 2.5 Statistical Analysis

Statistical analysis of data was performed with GraphPad Prism 5.0 (GraphPad Software, San Diego, CA, USA) and SPSS 23.0. Graphs were generated with the same softwares.

In order to evaluate data distribution, Shapiro-Wilk and one-sample Kolmogorov-Smirnov with Lilliefors correction tests were used. If data exhibited normal distribution for continuous variables, the values were expressed as means with standard deviation (SD). For non-gaussian data, the median with interquartile 25%/75% range [IQR] was used, and for categorical variables as numbers (percentage), absolute and relative frequencies were used. *T*-test, median, two-sample Kolmogorov-Smirnov, and Mann-Whitney U tests were used to compare the variance/mean/median differences between groups. Wilcoxon signed-ranks test (two-related samples) was used for differences between time points (*ex vivo*/day 7/day 14). The percentage changes of immune cell subsets were calculated from absolute numbers in comparison to baseline. *P*-values were considered significant when equal to or lower than 0.05: *P ≤ 0.05, **P ≤ 0.01, ***P ≤ 0.005, ****P ≤ 0.0001.

The correlation between two sets of data was assessed by Spearman’s or Pearson’s test, depending on data distribution and type. Strength of correlation was classified as null/very weak (|*r|* < 0.25), acceptable (0.25 ≤ |*r|* < 0.5), moderate (0.5 ≤ |*r|* < 0.75), or very good (|*r|* ≥ 0.75). Statistical testing was performed at the two‐tailed α‐level of 0.05.

## 3 Results

Cellular analysis was performed for 34 RRMS patients and 17 HC. The RRMS group consisted of 32.35% males, while the control group was made up of 35.29% males (*p*>0.05). Mean ages were 35.9 years for the RRMS group and 33.2 years for the HC group (*p*>0.05).

### 3.1 T Cell Immunophenotype Shift From *Ex Vivo* to *In Vitro*

**Figure 3:**
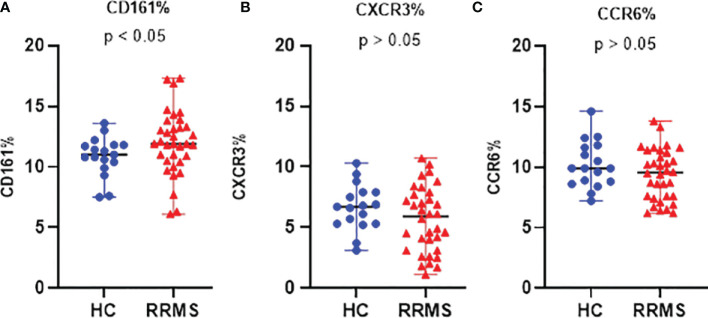
The proportion of *ex vivo* CD161 **(A)**, CXCR3 **(B)**, and CCR6 **(C)** positive cells in HC and RRMS groups. Each dot represents one individual subject and horizontal bars indicate median values. Shown *p*-values were calculated using the median test.

In *ex vivo* samples, the proportion of CD3+CD161+ cells was significantly increased in RRMS patients compared to HC (*p*<0.05) (**Figure 3A**).

**Figure 4:**
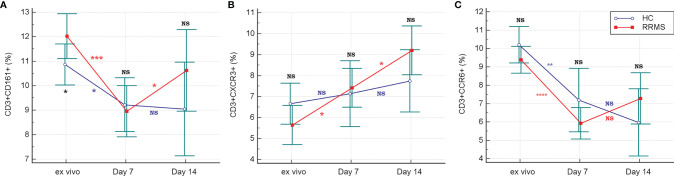
The evolution of CD3+ cells expressing CD161 **(A)**, CXCR3 **(B)** and CCR6 **(C)** receptors in *ex vivo* samples to 7 and 14 days in culture with rh-IL2. Significance levels are graphically represented as follows: NS, non significant, *p*>0.05, *p<0.05, **p<0.01, ***p<0.001, ****p<0.0001.

The percentage of *ex vivo* CD3+CXCR3+ (**Figure 3B**) and CD3+CCR6+ cells (**Figure 3C**) was not significantly different in RRMS patients compared to HC. Compared to *ex vivo*, the CD3+CD161+ cell subpopulation at day 7 *in vitro* was significantly decreased in both RRMS patients (p=0.0002) and HC subjects (p=0.013). Compared to day 7, the percentage of CD3+CD161+ cells at day 14 decreased slightly in cultures from HC subjects and increased in RRMS patients (p<0.05) (**Figure 4A**). A significant increase in the CD3+CXCR3+ cell subpopulation was found in RRMS patients after 7 and 14 days in culture, relative to *ex vivo* cells (*p*=0.018 and *p*=0.014, respectively). For HC, non-significant variations of CD3+CXCR3+ cell subpopulations were found after 7 and 14 days in culture (**Figure 4B**). A significant decrease in the CD3+CCR6+ population was observed in RRMS patients (p<0.0001) and HC (p=0.0075) after 7 days in culture, with no further significant changes noted at day 14 (**Figure 4C**).

**Figure 5:**
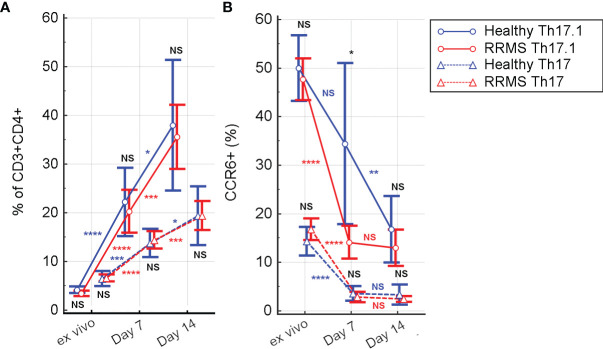
The evolution of Th17 (CD3+CD4+CD161+CXCR3−) and Th17.1 (CD3+CD4+CD161+CXCR3+) cell subpopulations **(A)** and of CCR6 positive Th17 and Th17.1 cells **(B)** from *ex vivo* to 7 and 14 days in culture with rh-IL2. Significance levels are graphically represented as follows: NS, non significant, *p*>0.05, *p<0.05, **p<0.01, ***p<0.001, ****p<0.0001.

CD3+CD4+CD161+CXCR3+ cells (Th 17.1) from HC showed a significant *in vitro* proliferation under rh-IL2 at day 7 relative to *ex vivo* samples (*p*<0.0001), associated with a sustained increase at day 14 (*p*=0.02). For RRMS subjects, Th17.1 proliferation was significant in the first 7 days (*p*<0.0001) and also between days 7 and 14 (*p*=0.0007) (**Figure 5A**). *In vitro* cultivation led to loss of CCR6 expression. In both Th17 and Th17.1 cell populations (CD3+CD4+CD161+CXCR3− and CD3+CD4+CD161+CXCR3+, respectively), the percentage of CCR6+ cells decreased over time. The Th17 population in both HC and RRMS subjects rapidly lost CCR6 expression from the *ex vivo* stage to day 7 (*p*<0.0001). Subsequently, from day 7 to day 14, the loss was non-significant. CCR6 cell surface expression decreased non-significantly on Th17.1 cells in HC from *ex vivo* to day 7, and significantly until day 14 (*p*=0.0013). In RRMS subjects, CCR6 was lost rapidly on Th17.1 and Th17 cells from *ex vivo* to day 7 (*p*<0.0001), but afterward was non-significant – results that were consistent with the evolution of CCR6+ expression on CD3+ cells (**Figures 5B**, **4C**).

In *ex vivo* samples, the percentage of Th17.1 cells was significantly lower than that of Th17 (for HC*, p*=0.0094 and for RRMS, *p*<0.0001). However, in rh-IL2 culture conditions, the Th17.1 subpopulation became predominant at day 7 (*p*=0.05 for HC and *p*=0.028 for RRMS) and day 14 (*p*=0.037 for HC and *p*=0.0009 for RRMS patients) (**Figure 5A**).

### 3.2 *Ex Vivo* Versus *In Vitro* Cytokine Production Capacity of Activated T Cells

**Figure 6:**
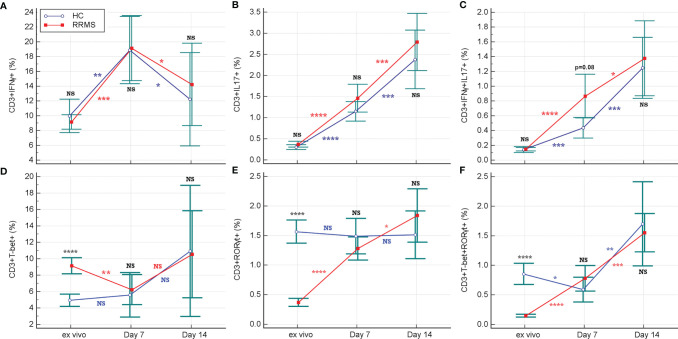
Changes in the proportion of CD3+IFNγ+ and CD3+TBet+ **(A, D)**, CD3+IFNγ+IL17+ and CD3+TBet+RORγt+ **(B, E)**, CD3+IL17+ and CD3+RORγt+ **(C, F)** cell subpopulations from *ex vivo* stage to days 7 and 14 of culture with rh-IL2. Significance levels are graphically presented as follows: NS, non significant, *p*>0.05,*p<0.05, **p<0.01, ***p<0.001, ****p<0.0001.

Flow cytometric analysis of the intracellular cytokine profile of CD3+ cells after short-term maximal activation revealed a significant increase in the percentage of CD3+IFNγ+ cells for HC and RRMS patients after 7 days in culture (*p*=0.0024 and *p*=0.0005, respectively), followed by a significant decrease at day 14 (*p*=0.013 for HC and *p*=0.015 for RRMS subjects) (**Figure 6A**). The proportion of IL17-producing cells also increased in HC and RRMS patients from *ex vivo* up to day 7 (both *p*<0.0001), and additionally until day 14 (*p*=0.0004 for HC and *p*=0.0006 for RRMS subjects) (**Figure 6B**). Double-positive CD3+IFNγ+IL17+ cells increased in HC and RRMS patients after 7 days in culture, relative to cells analyzed *ex vivo* (*p*<0.0001 for RRMS subjects and *p*=0.0001 for HC) and continued to increase until day 14 (*p*=0.0007 for HC and *p*=0.022 for RRMS subjects) (**Figure 6C**). There was a significant increase in the CD3+IFNγ+IL17+ and CD3+IL17+ cell subpopulations, which proliferated well after rh-IL2 activation *in vitro*, in both HC and RRMS subjects.

*Ex vivo* TBet-positive cells, which correspond to Th1 and Th17.1 phenotypes, were significantly increased in RRMS compared to HC (*p*<0.0001). On the contrary, in *ex vivo*, CD3+RORγt+ cells, which correspond to Th17 and Th17.1 phenotypes, were significantly decreased in RRMS patients (*p*<0.0001).

The proportion of CD3+ cells positive for TBet (which is a transcriptional regulator of IFNγ), had non-significant variations in the HC group and a weakly significant increase (*p*=0.036) in RRMS patients between *ex vivo* status and day 14 of culture. Considering the evolution of CD3+IFNγ+ and CD3+TBet+ cells, shown in **Figures 6A, D**, the significant increase in the CD3+CXCR3+ cell population (**Figure 4B**) was probably due to a Th17.1 subpopulation, which was more resistant and proliferative in response to *in vitro* activation with rh-IL2.

In addition to the increased proportions of CD3+IFNγ+IL17+ and CD3+IL17+ cell subpopulations, we observed an increase in the CD3+RORγt+ cell population from *ex vivo* to *in vitro* status at day 14, but only for RRMS patients. (*p*<0.0001 at day 7 and *p*=0.035 at day 14) (**Figure 6E** **)**. The analysis also revealed an increase in both transcription factors (double-positive CD3+TBet+RORγt+ cells) at day 14 for HC (*p*=0.0015) as well as for RRMS patients (*p*= 0.0004) (**Figure 6F** **)**.

### 3.3 The Effect of *In Vitro* Cladribine Exposure on T Cells

#### 3.3.1 Proliferation of Surviving T Cells After Cladribine Cytotoxic Action

For HC, after 48hrs of cladribine exposure, a survival index of 0.42 was calculated, while the survival index of unexposed cells was 0.89 (*p*=0.0001). After rh-IL2 activation, the proliferation index of HC cells significantly increased by day 7 (*p*<0.0001 for untreated cells and *p*<0.001 for cladribine-treated cells). From day 7 to 14, the proliferation of untreated cells decreased significantly (*p*=0.031), while cladribine-treated cells continued to proliferate.

**Figure 7:**
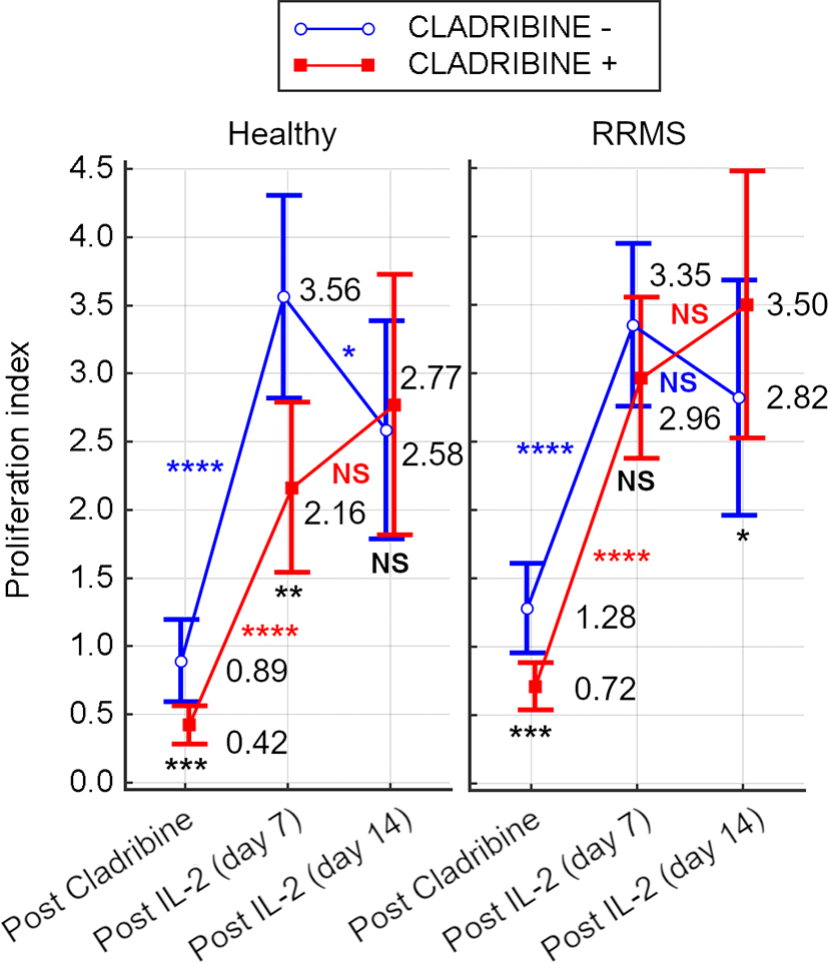
Survival index of cells from HC and RRMS subjects after cladribine exposure and proliferation index after 7 days or 14 days of rh-IL2 activation in cultures. Significance levels are graphically presented as follows: NS, non significant (p>0.05), *p<0.05, **p<0.01, ***p<0.001, ****p<0.0001.

For RRMS subjects, the survival index calculated after 48hrs of cladribine exposure was 0.72 for treated cells and 1.28 for untreated cells. In cultures including rh-IL2, the proliferation indices of RRMS significantly increased by day 7, similarly in both cladribine-treated and untreated cells (*p*<0.0001). From day 7 to 14, the proliferation of cladribine-treated cells increased non-significantly, while decreasing non-significantly for untreated cells. Under rh-IL2, the proliferation index of cladribine-treated cells increased at day 7 and again at day 14 while for untreated cells, the proliferation index increased only at day 7 (**Figure 7**).

#### 3.3.2 Immunomodulatory Effects of Cladribine

**Figure 8:**
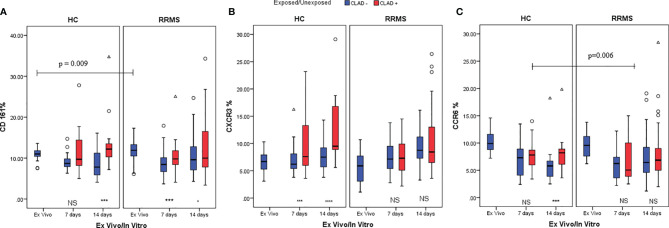
HC and RRMS CD3+CD161+ **(A)**, CD3+CXCR3+ **(B)** and CD3+CCR6+ **(C)** cell population changes at day 7 and day 14, after cladribine treatment, represented as boxplot (median with error bars). NS, non significant (*p* > 0.05) *P*-values were considered significant when lower than 0.05 (*p ≤ 0.05, ***p ≤ 0.005, ****p ≤ 0.0001).

The difference between unexposed and cladribine-exposed CD3+CD161+ cells in HC subjects was non-significant at day 7 (*p*=0.072). However, the difference was significant at day 14 (*p*=0.005) due to the increased proliferation of CD3+CD161+ cells in cladribine-exposed versus unexposed samples. Conversely, in RRMS subjects, the difference between the percentage of CD3+CD161+ cells in cladribine-exposed versus unexposed cells was higher at day 7 (*p*=0.005) but was lost until day 14 (*p*=0.015) (**Figure 8A**).

A sustained increase in CD3+CXCR3+ cell subpopulations from HC exposed to cladribine, compared to unexposed, was noted at day 7 (*p*=0.003) and 14 (*p*<0.0001). CD3+CXCR3+ cells exposed to cladribine had better survival and proliferation profiles only in HC. CD3+CXCR3+ cells from RRMS subjects had similar survival and proliferation profiles at day 7 and day 14, regardless of exposure to cladribine (**Figure 8B**).

For CD3+CCR6+ cells from HC at day 7, a non-significant difference was found between unexposed and cladribine-exposed cells. However, due to the increased proliferation of exposed CD3+CCR6+ cells between day 7 and 14, this difference became significant at day 14 (*p*=0.002). In RRMS subjects, the percentages of CD3+CCR6+ cells were not significantly different between exposed and unexposed cells at 7 and 14 days. The loss of CCR6 expression was lower in HC than in RRMS patients treated with cladribine in the first week of culture (*p*=0.006) (**Figure 8C**).

**Figure 9:**
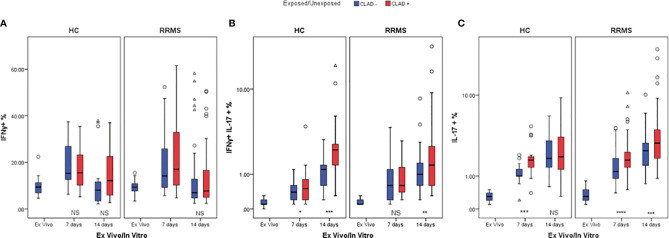
HC and RRMS CD3+IFNγ+ **(A)**, CD3+IFNγ+IL17+ **(B)**, and CD3+IL17+ **(C)** cell percentages changes with cladribine treatment at day 7 and 14 represented as boxplot (median with error bars). NS, non significant (p > 0.05) *P*-values were considered significant when lower than 0.05 (*p ≤ 0.05, **p ≤ 0.01, ***p ≤ 0.005, ****p ≤ 0.0001).

On day 7, unexposed and cladribine-exposed CD3+IFNγ+ cell subpopulations from the HC group proliferated similarly (*p*=0.098). On day 14, a lower percentage of CD3+IFNγ+ cells survived in culture. Unexposed cells exhibited especially poor survival. However, the difference between unexposed and cladribine-exposed CD3+IFNγ+ cell subpopulations remained non-significant. CD3+IFNγ+ cells from RRMS patients proliferated until day 7, with a weakly significant difference observed between exposed and unexposed cells (*p*=0.043). In the second week, the percentage of CD3+IFNγ+ cells decreased below *ex vivo* levels in both exposed and unexposed cells and the difference between them at day 14 was non-significant (**Figure 9A**). Cladribine exposure did not seem to significantly alter CD3+IFNγ+ cells proliferation in neither RRMS nor HC subjects.

HC CD3+IFNγ+IL17+ cells exposed to cladribine survived and proliferated better than unexposed cells until day 7 (*p*=0.036) and continued to fare significantly better (*p*=0.002) until day 14. For RRMS patients, the percentages of CD3+IFNγ+IL17+ cells also increased up to day 14 compared to *ex vivo* but were significantly different only at day 14 (*p*=0.006) (**Figure 9B**).

Cladribine-exposed CD3+IL17+ cell subpopulations exhibited greater survivability and proliferation than unexposed cells at day 7, for both HC (p=0.002) and RRMS subjects (p=0.0001). However, on day 14, a significant increase of exposed versus unexposed CD3+IL17+ cells was observed only in RRMS subjects (*p*=0.004) (**Figure 9C**).

#### 3.3.3 Secreted Cytokine Profile Changes in Response to Cladribine Treatment

The cytokine profile measured from culture media of TCR-stimulated cells revealed a significant decrease in IFNγ secreted by HC cells from the *ex vivo* stage to day 7 (*p*=0.001) and day 14 (*p*=0.002) of culture. For RRMS patients, a significant decrease in IFNγ secretion was also observed from the *ex vivo* stage to day 7 and day 14 (both *p*<0.0001). A similar decrease in IL17 secretion was measured from the *ex vivo* stage until day 7 (*p*=0.005 for HC and *p*<0.0001 for RRMS) and day 14 (*p*=0.001 for HC and p<0.0001 for RRMS). A general trend of IFNγ and IL17 secretion recovery is observed up to day 14 in both RRMS and HC subjects, regardless of cladribine exposure.

**Figure 10:**
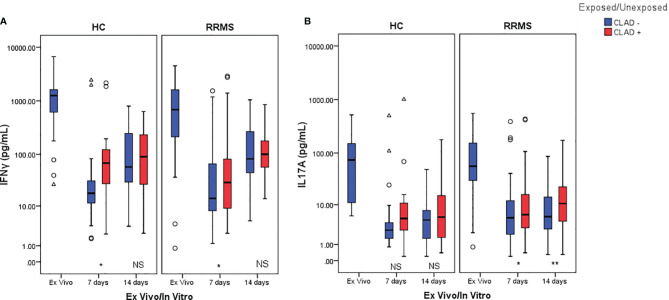
Changes in IFNγ **(A)** and IL17 **(B)** secretion profiles after cladribine exposure represented as boxplot (median with error bars). NS, non significant (p > 0.05) *P*-values were considered significant when lower than 0.05 (*p ≤ 0.05, **p ≤ 0.01).

IFNγ secretion from cladribine-treated cells significantly increased at day 7 relative to untreated cells (*p*=0.049 for HC and *p*=0.013 for RRMS), but at day 14 the differences were no longer significant (*p >*0.05) (**Figure 10A**). IL17 secretion from cladribine-treated vs untreated cells increased non-significantly for HC at day 7 and 14, but increased significantly for RRMS at both time points (*p*=0.027 at day 7 and *p*=0.007 at day 14) (**Figure 10B**).

## 4 Discussion

### 4.1 The Surface T Cell Phenotype Shifts From *Ex Vivo* to *In Vitro* Status

T cells involved in MS pathogenesis express a combination of encephalitogenic molecules from cells with Th1 and Th17 phenotypes, eliciting CNS inflammation (32). Our study first highlighted the presence of CD161, CXCR3, and CCR6 on the surface of T cells in *ex vivo* samples, which is expected to mirror the *in vivo* status of the cells quite accurately. The *ex vivo* T cell phenotype was compared with the phenotypic profile of *in vitro* rh-IL2-activated cells in medium-term cultures that are often used to study the effect of therapeutic molecules in the pre-clinical phases of trials.

As described by Fergusson et al. (33), the C-type lectin CD161 binds lectin-like transcript 1 (LLT1) expressed by both activated antigen presenting cells and lymphocytes, leading to increased IFNγ production in T lymphocytes. CD161 is considered a hallmark of Th17 cells and has been consistently associated with a memory phenotype in the adult circulation. Also, CD161 is involved in transendothelial migration in the absence of chemotactic stimuli by binding to acidic oligosaccharides on the endothelial cell surface (33). IL17 knockout mice exhibit delayed onset, reduced severity, and early recovery of experimental autoimmune encephalomyelitis (EAE), the murine model of MS. In humans, MS patients show increased IL17 levels (34), infiltration of CD3+IL17+ cells within MS brain lesions (35), and a significantly higher percentage of peripheral blood CD3+CD8+CD161^high^ cells compared to healthy subjects (15). Taken together, data from the literature provides sufficient evidence that CD161 is involved in MS pathogenesis in ways that are yet to be fully elucidated. As expected, in our study, a significantly higher percentage of CD3+CD161+ cells was observed in the *ex vivo* samples from RRMS patients compared to HC (**Figures 3A**, **4A**). Given that RRMS patients included in this study had no evidence of disease activity at the time of blood sampling, this finding suggests that increased CD3+CD161+ cell levels may serve as a potential biomarker for differentiating healthy individuals not only from relapsing MS patients but also from RRMS patients with no current evidence of disease activity. By the same logic, it could be speculated that an increased level of CD3+CD161+ cells (or an unexplained increase from the baseline) in yet undiagnosed future MS patients, may predict an upcoming clinically isolated syndrome as a first episode of MS. However, this is a speculation that remains to be addressed in future research. Regarding the *in vitro* evolution of CD3+CD161+ cells, the significant difference seen *ex vivo* between RRMS patients and HC is lost *in vitro*, with only an apparent recovery of RRMS CD3+CD161+ cells at day 14. Therefore, we consider that CD161 is not a suitable T cell marker for monitoring medium-term cultures using the activation protocol described in this paper.

As shown by Groom et al. (36), after initial T cell entry into the CNS, a subsequent inflow of T cells is mediated by CXCR3 (an interferon-inducible chemokine receptor). CXCR3 is associated with Th1 and CCR6+ Th1 cell phenotypes. CXCR3 binds three chemokines: CXCL9 (also known as MIG, monokine induced by gamma-interferon), CXCL10 (IP-10, interferon-induced protein of 10kDa), and CXCL11 (I-TAC, interferon-inducible T cell alpha chemoattractant), triggering the entry of CXCR3+ cells into the brain. Our analysis showed that the proportion of CD3+CXCR3+ cells from RRMS patients continued to significantly increase from *ex vivo* to 7 and then to 14 days in culture, while HC CD3+CXCR3+ cells increased non-significantly between these three time points (**Figures 3B**, **4B**). Despite that, there was no significant difference between HC and RRMS CD3+CXCR3+ cells at neither time point. Since CXCR3 is an inflammation-driven, interferon-inducible molecule, this lack of significant difference *ex vivo* may too be related to the lack of evident disease activity in our RRMS patients at the time of blood sampling. Also, the continuous increase of this cell population *in vitro* is probably caused by increased levels of CD3+IL17+ and CD3+IFNγ+IL17+ cells, the two pathogenic populations that are the focus of this study (see similar patterns for **Figures 4B**, **5A**, **6B, C**).

According to Restorick et al. (37) and Lee et al. (38), CCR6 (chemokine receptor 6) binds CCL20 (chemokine ligand 20), and this CCR6-CCL20 axis is required for the initial wave of T cells entering the CNS. CCR6+ cells are the first cells recruited across the choroid plexus into the CNS, driving the activation of the endothelial barrier and reducing barrier integrity in the first phase of MS disease. It can be presumed that the gradual *in vitro* loss of CD3+CCR6+ (**Figure 4C**), CCR6+ Th17 and Th17.1 cells (**Figure 5B**) seen in this study was caused by the absence of CCL20 stimulation.

In summary, *ex vivo* levels of CD3+CD161+ cells are significantly higher in RRMS patients without evidence of disease activity, and may therefore have practical applications. In turn, *ex vivo* levels of CD3+CXCR3+ and CD3+CCR6+ cells are not significantly different between HC and patients. However, the situation is likely to change in relapsing patients with ongoing disease activity. As elicited by our *in vitro* experimental model, CD161 and especially CCR6 do not seem to be suitable T cell markers for monitoring cell cultures. CD3+CXCR3+ cell percentages however seem to increase significantly in relation to pathogenic CD3+IL17+ and CD3+IFNγ+IL17+ cell populations.

### 4.2 *Ex Vivo* Versus *In Vitro* Cytokine Production Capacity of Maximally Activated T Cells

A peak of CD3+ cells with maximal IFNγ-producing capacity was identified at day 7 in culture for both HC and RRMS after rh-IL2 induction. Therefore, during the second week in culture, a resumption of TBet synthesis likely occurred and the percentage of CD3+TBet+ cells stabilized. As Ross et al. (39) described, in CD3+ cells activated with rh-IL2, TBX21 gene expression is triggered, followed by TBet transcription factor and IFNγ synthesis *via* a STAT5-dependent mechanism. CXCR3 synthesis is also driven by TBet (36), and IL2 promotes the proliferation of cells with Th1 phenotype (39).

Cell culture conditions determined a similar evolution pattern of CD3+IFNγ+ cells in both RRMS and HC subjects. Also, cladribine exposure does not seem to alter the proliferation of CD3+IFNγ+ cells.

*In vitro* rh-IL2 activation generated a significant increase in the CD3+IFNγ+IL17+ cell subpopulation for HC and RRMS subjects which, simultaneous with the evolution of CD3+CD161+ and CD3+CXCR3+ cells, was related to CD3+TBet+RORγt+ signaling at day 7 and day 14 of culture.

A significant increase in CD3+IL17+ cells was observed in HC and RRMS subjects. The number of CD3+RORγt+ cells remained constant for HC but increased in RRMS patients, which could also explain the evolution of CD3+CD161+ cells during the 14 days in culture. This evolution could have been a consequence of the proliferation of cells with a Th17 phenotype that also share differing degrees of Th1 features. The heterogeneous group of Th17 cells *in vitro* activated with rh-IL2 were found to exhibit good survivability and proliferation.

According to van Langelaar et al. (10), the heterogeneous cellular Th17 phenotype can be comprised of high (CCR6+CXCR3−) and low (CCR6+CXCR3+) IL17A-producers, that is CCR6+CXCR3−CCR4+IFNγ+IL17^high^ Th17 cells, CCR6+CXCR3+CCR4−IFNγ+IL17^low^ Th17.1 cells, and CCR6+CXCR3+CCR4+IFNγ+IL17^dim^ Th17 double-positives cells. Th17.1 (Th1-like Th17) cells have a Th17 origin and are characterized by IL17 and IFNγ co-expression (due to co-expression of transcription factors TBet and RORγt) and the presence of CCR6 and CXCR3, with or without CCR4 expression on the cell surface. Th17.1 cells also showed CD161 expression, as an ex-Th17 phenotype, but with distinct pro-inflammatory Th1 features due to the co-expression of VLA-4, CD161, TBet, RORγt, IFNγ, IL17 and GM-CSF (10). Th17.1 cells are recruited to the CNS to mediate early MS disease activity and are predominantly accumulated in the blood of relapse-free patients (10). As Kalra et al. (5) demonstrated, this group shares features of Th17 and Th1 cells, but also contains non-classical Th1 cells (known as ex-Th17 or non-conventional Th1 cells), which are significantly more numerous in the CSF as compared to the peripheral blood. These IFNγ-secreting Th17 cells express TBX21, CCR6 and CXCR3 surface markers and RORγt, but are characterized by the absence of Th17-associated cytokines.

Acquaviva et al. (40) showed that the gene expression of both IL17 and C-type lectin CD161, controlled by the transcription factor RORγt, is not only a feature of Th17 cells but also of a particular CD3+CD8+ cell type. Hinks and Zhang (41) described CD8+CD161^high^ cells, known as mucosal-associated invariant T (MAIT) cells. Fergusson et al. (33) showed that 10% of MAIT cells display an upregulated expression of RORγt and CCR6, representing a Tc17 cell type with diminished cytotoxic potential due to a reduced amount of the Tbox transcription factor, Eomesodermin, IFNγ, and granzyme B in comparison with CD3+CD8+ effector cells. The frequency of CD3+CD8+CD161+ lymphocytes decreased in blood but increased in the inflamed site by infiltrating MS lesions. Seventy to eighty percent of CD3+CD8+ cells from active MS lesions are known to produce IL17 (40). Patients with MS have a significantly higher percentage of peripheral blood CD3+CD8+CD161^high^ cells, which secrete more IL17 than in healthy individuals. Furthermore, IFNγ and IFNγ/IL17 co-secreting CD3+CD8+ cells were identified, expressing intermediate or high CD161 levels, while CD3+CD4+ cells express intermediate levels of CD161 (40).

In summary, this study enabled the emphasis to be placed on CD3+ cell subpopulations involved in MS pathology. More specific, the proposed *in vitro* experimental protocol has proven very efficient at selecting pathogenic Th17 and especially Th17.1 populations (**Figure 5A**), and also pathogenic secretory CD3+ cell populations such as CD3+IL17+ and CD3+IFNγ+IL17+ (**Figures 6B, C**). We conclude that the protocol is appropriate for personalized *in vitro* T cell profile assessment by short- and medium-term activation and for the *in vitro* investigation of the effect of therapeutic drugs on such selected populations. One limitation of the present study was the lack of characterization of CD3+CD8+ cells and their specific contribution to the evolution of CD3+ cell subpopulations in culture with rh-IL2.

### 4.3 The Effect of *In Vitro* Cladribine Exposure on T Cells

Heterogeneous brain-infiltrating lymphocyte subsets are known to contribute to MS pathogenesis (32). A better knowledge of the particular phenotype shift and proliferation capacity of lymphocyte subsets in response to DMDs can help in developing a predictive algorithm of responsiveness in order to tailor the best therapy for each MS patient. Characterization of immune cell alterations occurring during the disease course and in response to treatment may support a better understanding of MS pathogenesis and the mechanism of action of DMDs (42).

#### 4.3.1 Real-World Data on Cladribine Treatment for MS

Published in 2010, CLARITY was the first clinical trial on cladribine treatment for RRMS patients (43). This Phase III randomised controlled clinical trial demonstrated that treatment with oral cladribine significantly reduces the relapse rate, the risk of disability progression, and MRI measures of disease activity in RRMS patients (43). Subsequent clinical trials, clinical trials extensions, trial cohort follow-ups, and other studies have confirmed cladribine as a potent DMD for MS patients, with therapeutic efficiency lasting between 2-5 years or even up to 8 years in some individuals (29, 42, 44–49). Other ongoing clinical trials are aiming to further establish clear therapeutic indications for MS patients in various stages of the disease and clinical contexts (50).

Cladribine has been approved in the EU in 2017 and is currently indicated mostly for the treatment of patients with highly active RRMS. Regarding cladribine’s efficacy and real-world applications compared to other drugs, a Class III evidence, CLARITY/i-MuST propensity score matched study on RRMS patients showed that cladribine-treated patients had: lower annualized relapse rates compared with interferon, glatiramer acetate, or dimethyl fumarate; similar annualized relapse rates compared to fingolimod; and higher annualized relapse rates compared to natalizumab (51). Moreover, the beneficial effect of oral cladribine was generally higher in patients with high disease activity (51). Similar results were observed in a cohort of RRMS patients where cladribine was proven superior to interferon, similar to fingolimod, and inferior to natalizumab (52). Similarly, a recent publication suggests that oral cladribine is significantly more effective in achieving NEDA-3 (no evidence of disease activity-3, a composite endpoint comprising 3 measurements of disease activity: lack of clinical relapse, lack of confirmed disability progression, and no disease activity on MRI) than dimethyl fumarate and teriflunomide, but has similar efficacy compared with fingolimod (53). According to a network meta-analysis regarding the effects of RRMS drugs on annualized relapse rates and confirmed disability progression in patients with active and highly active RRMS, cladribine was superior to glatiramer acetate and interferon, but similar to dimethyl fumarate and fingolimod (54). In the same network meta-analysis, when compared with other DMDs, cladribine was ranked 4^th^ on a scale of efficacy for reduction of annualized relapse rate, after alemtuzumab, natalizumab, and ocrelizumab (54). However, regarding confirmed disease progression for 6 months, NEDA, and overall adverse event risk, oral cladribine did not differ significantly from most alternative DMDs (54). A recent study on 270 MS patients from Germany, reported that patients switching from natalizumab to cladribine tablets were prone to re-emerging disease activity (55). Similarly, recent evidence from patients switching from natalizumab to other DMDs shows that cladribine has a higher risk for relapses and MRI activity than ocrelizumab, but similar to rituximab (56). Regarding confirmed disability progression, there was no difference between patients switching from natalizumab to either cladribine, ocrelizumab, or rituximab (56). Overall, cladribine seems to be a safe exit strategy in natalizumab-treated RRMS patients at high risk of progressive multifocal leukoencephalopathy (56).

All things considered, there’s an increasing body of evidence from clinical trials and large studies in support of cladribine as a safe and efficient DMD in RRMS. However, despite these evidences and its superiority over other DMDs, the short- and long-term effects of cladribine on cells of the specific immune system are yet to be fully elucidated. Risk assessment is required before initiating cladribine therapy in MS patients, especially due to its lymphocyte-depleting effect which can cause severe lymphopenia. Moreover, cladribine treatment should ideally be initiated only for individuals responsive to this DMD. Hence, we aimed to assess the effect of cladribine on T cells phenotype as a means towards achieving the goal of precision medicine in RRMS patients.

#### 4.3.2 The Cytotoxic Effect of Cladribine

As a result of the cytotoxic effect of cladribine, the initial T cell proliferation index after 48h of cladribine exposure was lower for treated cells from both HC and RRMS patients, compared to untreated cells. However, the median proliferation index was higher in RRMS patients compared to HC (untreated: 1.28 vs 0.89; treated: 0.72 vs 0.42; see **Figure 7**). These differences suggest that T cells of RRMS patients tend to proliferate (as opposed to T cells of HC) and are also more resistant to cladribine compared to T cells of HC. In both HC and RRMS, only treated T cells proliferated continuously until day 14, suggesting that T cells resistant to cladribine also display a consistent proliferative behavior. Given the meteoric rise of Th17 and especially Th17.1 *in vitro* (**Figure 5A**), it is safe to assume that these two pathogenic populations share a certain degree of cladribine resistance and proliferative behavior.

#### 4.3.3 The Immunomodulatory Effect of Cladribine

Cellular subpopulations like Th17 and Th17.1 are very rare in the peripheral blood. Secretory profile assessment of T cells lineage using intracellular cytokines *in vitro* experimental protocol has been proven to be very efficient in the analysis of these T subpopulations. In cultures exposed to cladribine, CD3+CD161+ cells from HC were depleted until day 7, as in untreated cells; however, the exposed HC CD3+CD161+ cells proliferated up to day 14, which led to a significant difference between treated and untreated cells (**Figure 8A**). On day 7, there was a significantly higher percentage of treated CD3+CD161+ RRMS cells compared to untreated cells. The phenomenon of survival and proliferation after cladribine exposure continued for up to 14 days, further generating significantly higher percentages of CD161+ cells in cladribine treated cultures. CD3+CXCR3+ cells from HC were selected, surviving and proliferating well up to day 7 and day 14, with a significant difference observed between cladribine-treated and untreated cells. CD3+CXCR3+ cells from RRMS patients also proliferated well up to day 7 and day 14, with non-significant differences between cladribine-exposed and unexposed cell cultures (**Figure 8B**). The number of CD3+CCR6+ cells decreased only in the first week after cladribine exposure. In the second week, treated cells proliferated, highlighting a significant difference between untreated and cladribine-treated CD3+CCR6+ cells. The RRMS CD3+CCR6+ cell subpopulation was non-significantly modified in treated and untreated cell cultures on day 7 and day 14 (**Figure 8C**).

CD3+IFNγ+ cells from HC showed significant proliferation until day 7, followed by significant depletion at day 14, regardless of cladribine exposure. Cladribine-treated CD3+IFNγ+ cells from RRMS subjects also proliferated well during the first week, particularly compared to untreated cells. In the second week of culture, a similar depletion of untreated and treated cells was observed. The proliferation of the surviving CD3+IFNγ+IL17+cells from HC and RRMS subjects was more intense during the second week after cladribine exposure (**Figure 9B**). Cladribine-treated CD3+IL17+ cells from both HC and RRMS proliferated more than untreated cells at day 7, and only cladribine-treated RRMS CD3+IL17+ cells proliferated significantly more compared to untreated cells at day 14. Overall, CD3+CXCR3+, CD3+IL17+, and CD3+IFNγ+IL17+ are the populations with the highest proliferation after cladribine exposure. It appears that cladribine selects these T cell subpopulations, which survive and proliferate well. The recovery of CD161+ and CCR6+ cells at day 14 after cladribine exposure was also observed.

On day 7, IFNγ secretion in culture media from cladribine-treated cells was significantly higher than in untreated cells in both HC and RRMS subjects. On day 14, there is a non-significant difference between untreated and cladribine-treated cells. Given the much higher proportion of IFNγ single-positive cells (corresponding to Th1) relative to the T cell subpopulation double-positive for IFNγ and IL17, it can be presumed that Th1 cells make the largest contribution to IFNγ secretion. Indeed, similarly, the appearance of IFNγ single-positive cells has also been linked to IFNγ secretion in culture media. Exposure to cladribine led to a significant increase in CD3+IFNγ+ cells at day 7 only in RRMS patients and then had no effect at day 14. The levels of IFNγ secretion were different only at day 7, after which point there was no significant difference, regardless of cladribine exposure, in both HC and RRMS patients. These results, together with the evolution of the T cell phenotype *in vitro* at 7 and 14 days, indicate that a 14-day, double-stimulation *in vitro* experimental protocol is not necessarily needed, as data collected at day 14 is mainly redundant compared to data collected at day 7. Hence, we propose that, in view of precision medicine approaches to MS treatment, a 7-day, one-stimulation, standard experimental protocol be used for the induction of significant and measurable individual and disease-specific changes *in vitro*. Here, we tried to establish a relation between intracellular cytokine production and culture media cytokine levels. We emphasize that such efforts should be made in order to identify corresponding techniques that could replace costly and elaborate investigations like intracellular cytokine staining and multiparameter flow cytometry.

Despite the significant proliferation of cladribine-treated CD3+IL17+ cells at day 7, and of CD3+IFNγ+IL17+ cells at day 7 and day 14 of culture, IL17 secretion from HC cells was not different between untreated and treated cells at these time points. In RRMS patients, IL17 secretion from cladribine-treated cells was significantly higher at day 7 and day 14, in line with the proliferation of CD3+IL17+ and CD3+IFNγ+IL17+ cells exposed to cladribine.

As Korsen et al. (31) demonstrated, *in vitro* treatment with cladribine for 72 h, followed by PBMC culture for up to 58 days with IL2 and re-stimulation of surviving cells with anti-CD3/anti-CD28 antibodies or phytohemagglutinin, did not impair cell proliferation. Initial exposure of PBMCs from healthy subjects to cladribine only affected anti-inflammatory cytokine release. No significant changes were observed regarding IFNγ and IL17A secretion in culture media

An important issue is the influence of the different methods used for T cell activation. According to the cytokine profile determined by Olsen et al. (57), IFNγ and IL17 production were higher after T cell stimulation with PMA/ionomycin compared to anti-CD3/anti-CD28 mAbs. While T cell activation by anti-CD3/anti-CD28 mAbs is regarded as a physiological activation through the T-cell receptor complex (TCR), PMA/ionomycin activation triggers maximal cytokine production With appropriate concentration and stimulation time, PMA/ionomycin stimulation is useful to characterize cytokine production in poorly represented T cell subpopulations. While physiological activation may be the preferred option to assess the immune profile of T cells involved in MS, PMA/ionomycin activation provides more information on the immunomodulatory effect of DMDs such as cladribine. In this regard, the analysis of RRMS subjects, including those with outlier values, revealed an agreement between the results obtained by short maximal activation with PMA/ionomycin and by physiological activation with anti-CD3/anti-CD28 mAbs, only at day 7 in culture. On day 14, only the maximal activation provided significant results on the effect of cladribine (**Figures 9B, C**).

In a study recently published by Moser et al. (58), the immunological consequences of two cycles of therapy with oral cladribine for two years on 18 MS patients were investigated. The authors observed that CD3+CD4+ and CD3+CD8+ cells reached the lowest levels at month 15, followed by recovery at month 24. Memory and naïve T cytotoxic and helper cells were not altered by cladribine. Central memory and effector memory CD8+ cells, together with central memory Th17.1 (CD4+CD45RO+CCR7+CXCR3+CCR6^high^) cells, were depleted during the two-year period. Cladribine led to a depletion of Th1 and Th17.1 cells only at the end of year two, but no significant changes were noted in the cytokine expression profiles of Th1 and different Th17 subsets. In another study, Stuve et al. (59) analyzed the effects of 3.5 mg/kg cladribine administration after the first year of treatment. Cladribine intake led to a large reduction in B cells (approximately 80% at nadir), a moderate reduction in T cells (approximately 50% CD4+ and 40% CD8+ at nadir), and smaller reductions in NK cells (between 30% and 44% at nadir). The reductions were followed by a quick recovery towards baseline levels for CD16+/CD56+ and for CD19+ cells. The CD4+ and CD8+ T cells had a minimal and gradual recovery. Both memory and effector CD4+ T lymphocytes proportions decreased after treatment (63% and 54%, respectively, at nadir). CD4+ central memory remained lower at 48 weeks than at baseline, and CD4+ effector memory T cells were 16% higher at week 48 than at baseline.

All of the discussed findings raise the issue of cladribine action selectivity on different T cell subsets. Cladribine undergoes intracellular phosphorylation catalyzed by deoxycytidine kinase (DCK), generating active mononucleotide 2-chlorodeoxyadenosine 5’-triphosphate (2CdATP), which incorporates into DNA, blocking its synthesis and leading to cell death. DCK expression is variable in different types of lymphocytes, dependent on their degree of activation. Cells of the immune system present different sensitivities to cladribine, depending on the expression levels of DCK and two cytosolic 5-nucleotidases (5-NT) isoforms most involved in adenosine metabolism: higher DCK to 5-NT ratio means higher cladribine sensitivity (60). Among lymphocytes, DCK:5-NT is very high for B cells, intermediate for CD4+ T cells, and low for CD8+ T cells (60). The CLARITY clinical trial revealed that oral cladribine treatment was associated with a profound decline in lymphocyte counts from baseline (43). Although B cells are the most affected by cladribine, a gradual repopulation is seen toward the ends of both courses of treatment (27). On the other hand, T cell depletion was modest and dose-dependent, but sustained. Thus, Baker et al. hypothesized that the marked and long-lasting depletion of B cells is the key mechanism of action of cladribine in MS (27). However, given that major peripheral blood immune subsets such as CD4+/CD8+/CD19+ can not be used as biomarkers to predict disease activation following cladribine treatment, the authors also recognize that the mechanism of action of cladribine in MS is up to debate (27). It is likely that the pathogenic cells in MS are represented only by a minor population within any major cell subset. Therefore, cladribine’s beneficial effect in MS may be explained by either one or both qualitative and quantitative changes in one or multiple lymphocyte subsets. An in depth analysis of the many immune subsets may be the key to uncovering the mechanism of action of cladribine in MS.

All things considered, B cells have been so far the lymphocyte population most studied in MS. However, given the important role of T cells in the pathogenesis and progression of MS, we consider that a more in depth investigation of this lymphocyte population is needed in order to further clarify its role in MS. Therefore, we aimed to analyze both the quantitative (survival/proliferation index) and qualitative (immunophenotype) effects of *in vitro* cladribine exposure on T cells from RRMS patients in order to identify an algorithm able to differentiate between cladribine responders and non-responders. This endeavor represents a step towards precision medicine in MS.

**Figure 11:**
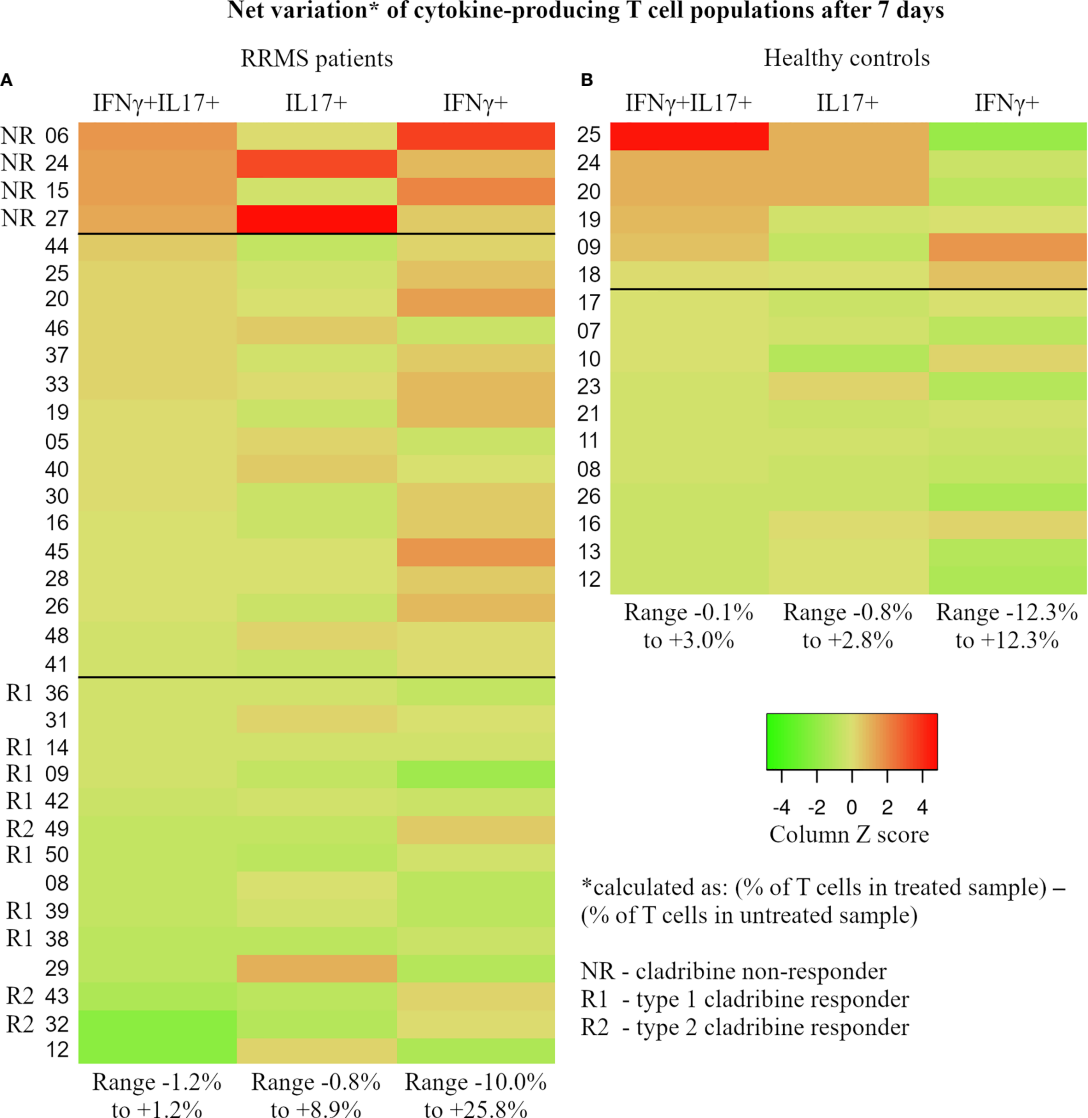
For each subject, the percentage of IFNγ+IL17+, IL17+, and IFNγ+ T cells was determined at day 7 through flow cytometry from paired cladribine-exposed and unexposed samples. The net effect of cladribine on the cytokine-producing populations was calculated for each subject as follows: (cytokine-producing population as % of T cells in the cladribine-exposed sample) – (the same cytokine-producing population as % of T cells in the unexposed sample). Calculated values reflect the true effect of cladribine and were used to generate the 2 heatmaps in this figure (**A** – RRMS patients, **B** – healthy donors). Based on the observed trends and patterns, RRMS patients were divided into 3 groups that may reflect each subject’s individual reaction to cladribine exposure: NR, non-responders (defined as net increase in all 3 cytokine-producing T cell populations after cladribine exposure), R1 – type 1 responders (defined as net decrease of all three cytokine-producing T cell populations), and R2 – type 2 responder (defined as net decrease of IFNγ+IL17+ and IL17+ T cells combined with net increase in IFNγ+ T cells). Note that HC and RRMS share the same color gradient. Also, the values are color coded based on Z score, while the range of absolute net % values for cell populations is presented at the bottom of each column.

The different responsiveness of particular lymphocyte subtypes to cladribine action, such as the sustained proliferation of cladribine-treated cells during two weeks in culture, was highlighted in our study. At the individual level, we assessed the net effect of cladribine on the evolution of cytokine-producing CD3+ cell populations after 7 days in culture (see **Figure 11**). Net variation is expressed as absolute percentage of cell population from the parent CD3+ cell population and is calculated as follows: (% value in treated samples) – (% value in untreated samples). Due to their role in the pathology of MS, CD3+IFNγ+IL17+ followed by CD3+IL17+ cell populations were the main focus of this assessment. Despite the apparent heterogeneity, **Figure 11A** shows a continuous and mainly unidirectional variation of cladribine effect on cytokine-producing CD3+ populations at day 7, defined by significant positive correlations between the (primary aggressive) CD3+IFNγ+IL17+ cell population and the CD3+IFNγ+ (Spearman’s r=0.662, p<0.0001) and CD3+IL17+ (Spearman’s r=0.356, p=0.038) cell populations. Interestingly, it should be noted that the top 10% of RRMS subjects on the list (**Figure 11A**; 4 RRMS patients: 06, 24, 15, and 27) presented systematically high values and were therefore classified as atypical for multiple parameters, including their response to short-term maximal activation with PMA/ionomycin/monensin, and to physiological stimulation. Given that, after cladribine exposure, all four subjects showed a significant net increase in all three cytokine-producing CD3+ cell populations (IFNγ+IL17+, IL17+, and IFNγ+), we consider these patients as possible drug-resistant or non-responders to cladribine. Inversely, most RRMS patients from the bottom 40% of the list (10 out of 14) show post-cladribine net variations of ≤ 0% for the CD3+IFNγ+IL17+ population and ≤ +0.5% for the CD3+IL17+ population. These negative net variations are caused by a lower prevalence of the pathogenic CD3+ cell populations in cladribine-treated samples compared to untreated samples, hinting at the suppressive role of cladribine on these pathogenic subsets. Hence, we consider these patients as possible responders to cladribine treatment. Moreover, based on the evolution of the CD3+IFNγ+ cell population, these responders can be further divided into two distinct types: type 1 (complete) responder (relative depletion of all 3 cell populations: CD3+IFNγ+IL17+, CD3+IL17+, and CD3+IFNγ+) and type 2 (incomplete) responder (relative depletion of CD3+IFNγ+IL17+ and CD3+IL17+ cells and relative selection of CD3+IFNγ+ cells). In summary, data analysis at the individual level with emphasis on the highs and lows of the same group, revealed that in some patients cladribine could be very effective in depleting IL17 and IFNγ secretory CD3+ cells, while in others, it may select highly reactive lymphocyte species, such as CD3+IFNγ+IL17+ and CD3+IL17+.

In HC, no similar pattern of responsiveness can be established. The bottom 50% of the list meets the main criterion of responsiveness used above for RRMS patients, that is a post-cladribine net variation of ≤ 0% for the CD3+IFNγ+IL17+ cells. Another similarity between HC and RRMS responders is that, except for one, all HC responders show negative net variation for CD3+IFNγ+ cells. However, in contrast to the established RRMS pattern, all of these HC responders show a positive net variation of CD3+IL17+ cells, ranging from 0.1% to 1.3%. This notable difference between HC responders, where net variations of CD3+IL17+ cells are all > 0%, and RRMS responders, where net variations of CD3+IL17+ cells are mainly ≤ 0%, is also supported by a negative correlation between the two cell populations in HC (Spearman’s r = -0.498, p=0.042). The HC subjects showing a non-responder behavior populate the upper 35% of the HC lot. However, once again in contrast to RRMS subjects, HC non-responders show mainly negative net variations for CD3+IFNγ+ cells whereas RRMS non-responders show highly positive net variations for CD3+IFNγ+ cells. A noteworthy case is the top HC non-responder which was an apparently healthy 35-year-old male who passed the exclusion criteria with a hsCRP level of 2.27 mg/L and a WBC count of 7.18×10^9^/L. The *ex vivo* levels of IFNγ and IL17A secreted in culture were as follows: IL17A 139.1 ng/mL (group range 5.8–519.8 ng/mL, median value 68.7 ng/mL, 4th largest value) and IFNγ 3671.3 ng/mL (group range 26.6–6666.9 ng/mL, median value 1167.5 ng/mL, 2nd largest value). Given the exaggerated response in *ex vivo* and *in vitro* experiments, we further investigated the levels of three inflammatory cytokines secreted in culture media: IL1β (3485 ng/mL), IL6 (17350 ng/mL), and TNFα (756 ng/mL). Given the described experimental behavior and cytokine levels together with the normal levels of hsCRP and WBC, we can only assume that this particular HC subject was going through the very early phase of an acute inflammation at the time of blood sampling.

A better knowledge of the particular phenotype shift and proliferation capacity of lymphocyte subsets in response to cladribine may help in developing a predictive algorithm of responsiveness in order to tailor the best therapy for each MS patient. This experimental model could be useful for precision therapy and to identify cladribine non-responders, to evaluate the behavior of Th1, Th17, Th17.1 cells from naïve MS patients in the presence of cladribine. As cladribine is an important member of immune reconstruction therapies, it is mandatory to determine what type of lymphocytes undergo this reconstruction process and what types are spared. In clinical practice there is no rebound of MS clinical or MRI activity even after recovery of T cell count, suggesting a degree of qualitative change after treatment with cladribine in the adaptive immune response (3, 61, 62).

A limitation of our study is patient-related since no relevant clinical data on the evolution of the investigated RRMS patients is available at the moment of writing. Therefore, only hypothetical, but no clear relations between the *ex vivo/in vitro* behavior of T cells and the clinical evolution of RRMS patients could be established. However, this is merely a temporal limitation as all RRMS patients included in this study are monitored periodically and, given the natural evolution of MS, more relevant clinical data will likely come to light over the following years.

The lately therapeutic developments seen in MS are unmatched in any field of neurology. Actually, modern treatment guidelines place the MS patient in the center of the treatment decision-making process for the improvement of MS management. There are nonresponders to DMDs among MS patients, but the amount of newly available DMDs on the market poses great challenges in selecting the right DMD for the right MS patients at the right moment. Hence, current interest and research targets to provide the tools and evidence for personalized medicine in MS. In the era of precision medicine, the decision to recommend treatment with cladribine to MS patients requires an understanding of cell responsiveness, which can be very different from patient to patient.

The experimental method we propose here could be the answer for the early prediction of the responsiveness of MS patient to cladribine. Additionally, this model may be extended to other DMDs.

## Data Availability Statement

The raw data supporting the conclusions of this article will be made available by the authors, without undue reservation.

## Ethics Statement

The studies involving human participants were reviewed and approved by the Committee for Ethical Research of the County Emergency Clinical Hospital of Târgu Mureș (decision no. 7,100/2018). The patients/participants provided their written informed consent to participate in this study.

## Authors Contribution

MD and RB conceived and designed the study. MD and DRM developed the methodologies. RB and LB provided patient samples and data. DM, DRM, AH and IBM analyzed data and performed experiments. MRG, LB and IBM performed statistical analysis of experimental data. MD and DRM analyzed data in the literature and drafted the manuscript. MD, AH and IBM technically revised the manuscript. MD and RB critically revised the manuscript for important intellectual content and approved the final version of the manuscript. All authors contributed to the article and approved the submitted version.

## Funding

This work was supported by Merck Regional Grant for Central and Eastern Europe Countries 2017 Research Project (No. 548/2018). The funder was not involved in the study design, collection, analysis, interpretation of data, the writing of this article or the decision to submit it for publication. Fees for open access publication are supported by “George Emil Palade” University of Medicine, Pharmacy, Science and Technology, Târgu Mureș, Romania.

## Conflict of Interest

The authors declare that the research was conducted in the absence of any commercial or financial relationships that could be construed as a potential conflict of interest.

## Publisher’s Note

All claims expressed in this article are solely those of the authors and do not necessarily represent those of their affiliated organizations, or those of the publisher, the editors and the reviewers. Any product that may be evaluated in this article, or claim that may be made by its manufacturer, is not guaranteed or endorsed by the publisher.

## References

[1] van Langelaar J Rijvers L Smolders J Van Luijn MM. B and T Cells Driving Multiple Sclerosis: Identity, Mechanisms and Potential Triggers. Front Immunol (2020) 11:760. doi: 10.3389/fimmu.2020.00760 32457742 PMC7225320

[2] Peterson LK Fujinami RS. Inflammation, Demyelination, Neurodegeneration and Neuroprotection in the Pathogenesis of Multiple Sclerosis. J Neuroimmunol (2007) 184(1-2):37–44. doi: 10.1016/j.jneuroim.2006.11.015 17196667 PMC1933528

[3] Balasa R Maier S Barcutean L Stoian A Motataianu A. The Direct Deleterious Effect of Th17 Cells in the Nervous System Compartment in Multiple Sclerosis and Experimental Autoimmune Encephalomyelitis: One Possible Link Between Neuroinflammation and Neurodegeneration. Rev Romana Med Laborator (2020) 28(1):9–17. doi: 10.2478/rrlm-2020-0005

[4] Durelli L Conti L Clerico M Boselli D Contessa G Ripellino P. T-Helper 17 Cells Expand in Multiple Sclerosis and Are Inhibited by Interferon-Beta. Ann Neurol (2009) 65:499–509. doi: 10.1002/ana.21652 19475668

[5] Kalra S Lowndes C Durant L Strange RC Al-Araji A Hawkins CP. Th17 Cells Increase in RRMS as Well as in SPMS, Whereas Various Other Phenotypes of Th17 Increase in RRMS Only. Mult Scler J Exp Transl Clin (2020) 6(1):2055217319899695. doi: 10.1177/2055217319899695 32064115 PMC6990617

[6] Annunziato F Cosmi L Santarlasci V Maggi L Liotta F Mazzinghi B. Phenotypic and Functional Features of Human Th17 Cells. J Exp Med (2007) 204(8):1849–61. doi: 10.1084/jem.20070663 PMC2118657 17635957

[7] Basdeo SA Cluxton D Sulaimani J Moran B Canavan M Orr C. Ex-Th17 (Nonclassical Th1) Cells Are Functionally Distinct From Classical Th1 and Th17 Cells and Are Not Constrained by Regulatory T Cells. J Immunol (2017) 198(6):2249–59. doi: 10.4049/jimmunol.1600737 28167631

[8] Boniface K Blumenschein WM Brovont-Porth K McGeachy MJ Basham B Desai B. Human Th17 Cells Comprise Heterogeneous Subsets Including IFN-γ–Producing Cells With Distinct Properties From the Th1 Lineage. J Immunol (2010) 185(1):679–87. doi: 10.4049/jimmunol.1000366 20511558

[9] Jones AP Kermode AG Lucas RM Carroll WM Nolan D Hart PH. Circulating Immune Cells in Multiple Sclerosis. Clin Exp Immunol (2017) 187:193–203. doi: 10.1111/cei.12878 27689339 PMC5217886

[10] van Langelaar J van der Vuurst de Vries RM Janssen M Wierenga-Wolf AF Spilt IM Siepman TA. T Helper 17.1 Cells Associate With Multiple Sclerosis Disease Activity: Perspectives for Early Intervention. Brain J Neurol (2018) 141(5):1334–49. doi: 10.1093/brain/awy069 29659729

[11] Kunkl M Frascolla S Amormino C Volpe E Tuosto L. T Helper Cells: The Modulators of Inflammation in Multiple Sclerosis. Cells (2020) 9(2):482. doi: 10.3390/cells9020482 PMC7072830 32093011

[12] Skulina C Schmidt S Dornmair K Babbe H Roers A Rajewsky K. Multiple Sclerosis: Brain-Infiltrating CD8+ T Cells Persist as Clonal Expansions in the Cerebrospinal Fluid and Blood. Proc Natl Acad Sci USA (2004) 101(8):2428–33. doi: 10.1073/pnas.0308689100 PMC356967 14983026

[13] Huber M Heink S Grothe H Guralnik A Reinhard K Elflein K. A Th17-Like Developmental Process Leads to CD8(+) Tc17 Cells With Reduced Cytotoxic Activity. Eur J Immunol (2009) 39(7):1716–25. doi: 10.1002/eji.200939412 19544308

[14] Salou M Garcia A Michel L Gainche-Salmon A Loussouarn D Nicol B. Expanded CD8 T-Cell Sharing Between Periphery and CNS in Multiple Sclerosis. Ann Clin Transl Neurol (2015) 2(6):609–22. doi: 10.1002/acn3.199 PMC4479522 26125037

[15] Annibali V Ristori G Angelini DF Serafini B Mechelli R Cannoni S. CD161(high)Cd8+T Cells Bear Pathogenetic Potential in Multiple Sclerosis. Brain (2011) 134(Pt 2):542–54. doi: 10.1093/brain/awq354 21216829

[16] Salou M Nicol B Garcia A Laplaud DA. Involvement of CD8(+) T Cells in Multiple Sclerosis. Front Immunol (2015) 6:604. doi: 10.3389/fimmu.2015.00604 26635816 PMC4659893

[17] Willing A Jäger J Reinhardt S Kursawe N Friese MA. Production of IL-17 by MAIT Cells Is Increased in Multiple Sclerosis and Is Associated With IL-7 Receptor Expression. J Immunol (2018) 200(3):974–82. doi: 10.4049/jimmunol.1701213 29298833

[18] Beltrán E Gerdes LA Hansen J Flierl-Hecht A Krebs S Blum H. Early Adaptive Immune Activation Detected in Monozygotic Twins With Prodromal Multiple Sclerosis. J Clin Invest (2019) 129(11):4758–68. doi: 10.1172/JCI128475 PMC6819125 31566584

[19] Denic A Wootla B Rodriguez M. CD8(+) T Cells in Multiple Sclerosis. Expert Opin Ther Targets (2013) 17(9):1053–66. doi: 10.1517/14728222.2013.815726 PMC3928018 23829711

[20] Wagner CA Roqué PJ Goverman JM. Pathogenic T Cell Cytokines in Multiple Sclerosis. J Exp Med (2020) 217(1):e20190460. doi: 10.1084/jem.20190460 31611252 PMC7037255

[21] Tillery EE Clements JN Howard Z. What’s New in Multiple Sclerosis? Ment Health Clin (2018) 7(5):213–20. doi: 10.9740/mhc.2017.09.213 PMC6007716 29955526

[22] Gajofatto A Benedetti MD. Treatment Strategies for Multiple Sclerosis: When to Start, When to Change, When to Stop? World J Clin Cases (2015) 3(7):545–55. doi: 10.12998/wjcc.v3.i7.545 PMC4517331 26244148

[23] Wingerchuk DM Weinshenker BG. Disease Modifying Therapies for Relapsing Multiple Sclerosis. BMJ (2016) 354:i3518. doi: 10.1136/bmj.i3518 27549763

[24] Rammohan K Coyle PK Sylvester E Galazka A Dangond F Grosso M. The Development of Cladribine Tablets for the Treatment of Multiple Sclerosis: A Comprehensive Review. Drugs (2020) 80(18):1901–28. doi: 10.1007/s40265-020-01422-9 PMC7708385 33247831

[25] Schreiber K Soelberg Sorensen P. Cladribine in the Treatment of Multiple Sclerosis. Clin Invest (2011) 1(2):317–26. doi: 10.4155/CLI.10.32

[26] Baker D Pryce G Herrod SS Schmierer K. Potential Mechanisms of Action Related to the Efficacy and Safety of Cladribine. Mult Scler Relat Disord (2019) 30:176–86. doi: 10.1016/j.msard.2019.02.018 30785074

[27] Baker D Herrod SS Alvarez-Gonzalez C Zalewski L Albor C Schmierer K. Both Cladribine and Alemtuzumab may Effect MS *via* B-Cell Depletion. Neurol Neuroimmunol Neuroinflamm (2017) 4(4):e360. doi: 10.1212/NXI.0000000000000360 28626781 PMC5459792

[28] Moser T Akgün K Proschmann U Sellner J Ziemssen T. The Role of TH17 Cells in Multiple Sclerosis: Therapeutic Implications. Autoimmun Rev (2020) 19(10):102647. doi: 10.1016/j.autrev.2020.102647 32801039

[29] Leist TP Comi G Cree BA Coyle PK Freedman MS Hartung HP. Effect of Oral Cladribine on Time to Conversion to Clinically Definite Multiple Sclerosis in Patients With a First Demyelinating Event (ORACLE MS): A Phase 3 Randomised Trial. Lancet Neurol (2014) 13(3):257–67. doi: 10.1016/S1474-4422(14)70005-5 24502830

[30] Șerban GM Mănescu IB Manu DR Dobreanu M. Optimization of a Density Gradient Centrifugation Protocol for Isolation of Peripheral Blood Mononuclear Cells. Acta Med Marisiensis (2018) 64(2):83–90. doi: 10.2478/amma-2018-0011

[31] Korsen M Bragado Alonso S Peix L Bröker BM Dressel A. Cladribine Exposure Results in a Sustained Modulation of the Cytokine Response in Human Peripheral Blood Mononuclear Cells. PloS One (2015) 10(6):e0129182. doi: 10.1371/journal.pone.0129182 26086440 PMC4472752

[32] Segal BM. The Diversity of Encephalitogenic CD4+ T Cells in Multiple Sclerosis and Its Animal Models. J Clin Med (2019) 8(1):120. doi: 10.3390/jcm8010120 PMC6352150 30669462

[33] Fergusson JR Fleming VM Klenerman P. CD161-Expressing Human T Cells. Front Immunol (2011) 2:36. doi: 10.3389/fimmu.2011.00036 22566826 PMC3342360

[34] Lock C Hermans G Pedotti R Brendolan A Schadt E Garren H. Gene-Microarray Analysis of Multiple Sclerosis Lesions Yields New Targets Validated in Autoimmune Encephalomyelitis. Nat Med (2002) 8(5):500–8. doi: 10.1038/nm0502-500 11984595

[35] Tzartos JS Friese MA Craner MJ Palace J Newcombe J Esiri MM. Interleukin-17 Production in Central Nervous System-Infiltrating T Cells and Glial Cells Is Associated With Active Disease in Multiple Sclerosis. Am J Pathol (2008) 172(1):146–55. doi: 10.2353/ajpath.2008.070690 PMC2189615 18156204

[36] Groom JR Luster AD. CXCR3 in T Cell Function. Exp Cell Res (2011) 317(5):620–31. doi: 10.1016/j.yexcr.2010.12.017 PMC3065205 21376175

[37] Restorick SM Durant L Kalra S Hassan-Smith G Rathbone E Douglas MR. CCR6+ Th Cells in the Cerebrospinal Fluid of Persons With Multiple Sclerosis are Dominated by Pathogenic Non-Classic Th1 Cells and GM-CSF-Only-Secreting Th Cells. Brain Behav Immun (2017) 64:71–9. doi: 10.1016/j.bbi.2017.03.008 PMC5490506 28336414

[38] Lee AYS Körner H. The CCR6-CCL20 Axis in Humoral Immunity and T-B Cell Immunobiology. Immunobiology (2019) 224(3):449–54. doi: 10.1016/j.imbio.2019.01.005 30772094

[39] Ross SH Cantrell DA. Signaling and Function of Interleukin-2 in T Lymphocytes. Annu Rev Immunol (2018) 36:411–33. doi: 10.1146/annurev-immunol-042617-053352 PMC6472684 29677473

[40] Acquaviva M Bassani C Sarno N Dalla Costa G Romeo M Sangalli F. Loss of Circulating CD8+ CD161high T Cells in Primary Progressive Multiple Sclerosis. Front Immunol (2019) 10:1922. doi: 10.3389/fimmu.2019.01922 31474991 PMC6702304

[41] Hinks TSC Zhang XW. MAIT Cell Activation and Functions. Front Immunol (2020) 11:1014. doi: 10.3389/fimmu.2020.01014 32536923 PMC7267072

[42] Giovannoni G Soelberg Sorensen P Cook S Rammohan K Rieckmann P Comi G. Safety and Efficacy of Cladribine Tablets in Patients With Relapsing Remitting Multiple Sclerosis: Results From the Randomized Extension Trial of the CLARITY Study. Mult Scler (2018) 24:1594–604. doi: 10.1177/1352458517727603 28870107

[43] Giovannoni G Comi G Cook S Rammohan K Rieckmann P Soelberg Sørensen P. CLARITY Study Group. A Placebo-Controlled Trial of Oral Cladribine for Relapsing Multiple Sclerosis. N Engl J Med (2010) 362(5):416–26. doi: 10.1056/NEJMoa0902533 20089960

[44] Thompson AJ Baranzini SE Geurts J Hemmer B Ciccarelli O. Multiple Sclerosis. Lancet (2018) 391(10130):1622–36. doi: 10.1016/S0140-6736(18)30481-1 29576504

[45] Montalban X Leist TP Cohen BA Moses H Campbell J Hicking C. Cladribine Tablets Added to IFN-β in Active Relapsing MS: The ONWARD Study. Neurol Neuroimmunol Neuroinflamm (2018) 5(5):e477. doi: 10.1212/NXI.0000000000000477 30027104 PMC6047834

[46] Cook S Leist T Comi G Montalban X Giovannoni G Nolting A. Safety of Cladribine Tablets in the Treatment of Patients With Multiple Sclerosis: An Integrated Analysis. Mult Scler Relat Disord (2019) 29:157–67. doi: 10.1016/j.msard.2018.11.021 30885374

[47] Patti F Visconti A Capacchione A Roy S Trojano M CLARINET-MS Study Group. Long-Term Effectiveness in Patients Previously Treated With Cladribine Tablets: A Real-World Analysis of the Italian Multiple Sclerosis Registry (CLARINET-Ms). Ther Adv Neurol Disord (2020) 13:1756286420922685. doi: 10.1177/1756286420922685 32587633 PMC7294475

[48] Lizak N Hodgkinson S Butler E Lechner-Scott J Slee M McCombe PA. Real-World Effectiveness of Cladribine for Australian Patients With Multiple Sclerosis: An MSBase Registry Substudy. Mult Scler (2021) 27(3):465–74. doi: 10.1177/1352458520921087 PMC7897790 32530363

[49] Moccia M Lanzillo R Petruzzo M Nozzolillo A De Angelis M Carotenuto A. Single-Center 8-Years Clinical Follow-Up of Cladribine-Treated Patients From Phase 2 and 3 Trials. Front Neurol (2020) 11:489. doi: 10.3389/fneur.2020.00489 32625161 PMC7311570

[50] Miravalle AA Katz J Robertson D Hayward B Harlow DE Lebson LA. CLICK-MS and MASTER-2 Phase IV Trial Design: Cladribine Tablets in Suboptimally Controlled Relapsing Multiple Sclerosis. Neurodegener Dis Manage (2021) 11(2):99–111. doi: 10.2217/nmt-2020-0059 33517769

[51] Signori A Saccà F Lanzillo R Maniscalco GT Signoriello E Repice AM. Cladribine vs Other Drugs in MS: Merging Randomized Trial With Real-Life Data. Neurol Neuroimmunol Neuroinflamm (2020) 7(6):e878. doi: 10.1212/NXI.0000000000000878 32801167 PMC7641098

[52] Kalincik T Jokubaitis V Spelman T Horakova D Havrdova E Trojano M. MSBase Study Group. Cladribine Versus Fingolimod, Natalizumab and Interferon β for Multiple Sclerosis. Mult Scler (2018) 24(12):1617–26. doi: 10.1177/1352458517728812 28857680

[53] Bartosik-Psujek H Kaczyński Ł Górecka M Rolka M Wójcik R Zięba P. Cladribine Tablets Versus Other Disease-Modifying Oral Drugs in Achieving No Evidence of Disease Activity (NEDA) in Multiple Sclerosis-A Systematic Review and Network Meta-Analysis. Mult Scler Relat Disord (2021) 49:102769. doi: 10.1016/j.msard.2021.102769 33516133

[54] Siddiqui MK Khurana IS Budhia S Hettle R Harty G Wong SL. Systematic Literature Review and Network Meta-Analysis of Cladribine Tablets Versus Alternative Disease-Modifying Treatments for Relapsing-Remitting Multiple Sclerosis. Curr Med Res Opin (2018) 34(8):1361–71. doi: 10.1080/03007995.2017.1407303 29149804

[55] Pfeuffer S Rolfes L Hackert J Kleinschnitz K Ruck T Wiendl H. Effectiveness and Safety of Cladribine in MS: Real-World Experience From Two Tertiary Centres. Mult Scler (2021) 12:13524585211012227. doi: 10.1177/13524585211012227 PMC8795224 33975489

[56] Zanghì A Gallo A Avolio C Capuano R Lucchini M Petracca M. Exit Strategies in Natalizumab-Treated RRMS at High Risk of Progressive Multifocal Leukoencephalopathy: A Multicentre Comparison Study. Neurotherapeutics (2021) 18(2):1166–74. doi: 10.1007/s13311-021-01037-2 PMC8423885 33844155

[57] Olsen I Sollid LM. Pitfalls in Determining the Cytokine Profile of Human T Cells. J Immunol Methods (2013) 390(1-2):106–12. doi: 10.1016/j.jim.2013.01.015 23416458

[58] Moser T Schwenker K Seiberl M Feige J Akgun K Haschke-Becher E. Long-Term Peripheral Immune Cell Profiling Reveals Further Targets of Oral Cladribine in MS. Ann Clin Transl Neurol (2020) 7(11):2199–212. doi: 10.1002/acn3.51206 PMC7664268 33002321

[59] Stuve O Soelberg Soerensen P Leist T Giovannoni G Hyvert Y Damian D. Effects of Cladribine Tablets on Lymphocyte Subsets in Patients With Multiple Sclerosis: An Extended Analysis of Surface Markers. Ther Adv Neurol Disord (2019) 12:1756286419854986. doi: 10.1177/1756286419854986 31244898 PMC6582297

[60] Voo VTF Butzkueven H Stankovich J O’Brien T Monif M. The Development and Impact of Cladribine on Lymphoid and Myeloid Cells in Multiple Sclerosis. Mult Scler Relat Disord (2021) 52:102962. doi: 10.1016/j.msard.2021.102962 33901971

[61] Comi G Cook S Giovannoni G Rieckmann P Sørensen PS Vermersch P. Effect of Cladribine Tablets on Lymphocyte Reduction and Repopulation Dynamics in Patients With Relapsing Multiple Sclerosis. Mult Scler Relat Disord (2019) 29:168–74. doi: 10.1016/j.msard.2019.01.038 30885375

[62] Balasa R Barcutean L Balasa A Motataianu A Roman-Filip C Manu D. The Action of TH17 Cells on Blood Brain Barrier in Multiple Sclerosis and Experimental Autoimmune Encephalomyelitis. Hum Immunol (2020) 81(5):237–43. doi: 10.1016/j.humimm.2020.02.009 32122685

